# Cytomegalovirus-positive Posner-Schlossman syndrome: to compare differences in retinal vessel area density between the affected and non-affected eye using optical coherence tomography angiography

**DOI:** 10.1007/s00417-023-06171-5

**Published:** 2023-07-22

**Authors:** Patricia Hülse, Emanuel Reitemeyer, Anne Rübsam, Uwe Pleyer, Anna-Karina B. Maier

**Affiliations:** Department of Ophthalmology, Charité–Universitätsmedizin Berlin, corporate member of Freie Universität Berlin, Humboldt Universität zu Berlin, and Berlin Institute of Health, Campus Virchow Klinikum, Augustenburger Platz 1, 13353 Berlin, Germany

**Keywords:** Cytomegalovirus, Glaucoma, OCT angiography, Posner-Schlossman syndrome, Uveitis, Vessel density

## Abstract

**Purpose:**

To analyse differences in the retinal microvasculature in eyes with cytomegalovirus (CMV)-positive Posner-Schlossman syndrome (PSS) compared to the non-affected eyes using optical coherence tomography angiography (OCTA).

**Methods:**

In this monocentric, observational prospective case series, 25 patients with unilateral CMV-positive PSS were included. We compared the vessel area densities (VAD) in the macula, optic disc, and peripapillary region in PSS-affected and non-affected eyes using OCTA. We compared the visual fields (VF) of the affected and healthy eyes of each patient. The mean deviation (MD) of the VF was analysed together with the retinal nerve fibre layer (RNFL) thickness to evaluate the strength of correlation with the VAD parameters.

**Results:**

The VAD of the peripapillary superficial vascular complex (SVC) is significantly reduced in CMV-positive PSS-affected eyes (46.1 ± 9.3% versus 50.1 ± 6.3%, *p* = 0.008, adjusted *p* = 0.048). The VAD of the deeper macular, papillary, and peripapillary layers showed no differences between the affected and non-affected eyes. The mean deviation and the retinal nerve fibre layer thickness had correlations with the VAD of the macula (*r* = 0.451, *p* = 0.001, *r* = 0.553, *p* < 0.001), the peripapillary SCV (*r* = 0.430, *p* = 0.002, *r* = 0.723, *p* < 0.001), and the papillary region (*r* = 0.512, *p* < 0.001, *r* = 0.292, *p* = 0.039). Patients receiving systemic antiviral therapy (SAT) showed better VAD of the peripapillary choriocapillary layer (*p* = 0.001, no therapy: 31.4 ± 1.9%, SAT: 35.0 ± 1.6%), and choroidal layer (*p* = 0.009, no therapy: 34.2 ± 0.3%, SAT: 36.3 ± 1.8%) compared to those with no SAT.

**Conclusion:**

A lower peripapillary VAD in the SVC might indicate vascular dysfunction as a sign of glaucomatous damage. SAT might have positive effects on the microcirculation in the deep retinal and choroidal layers.

**Trial registration:**

TRN: DRKS00028266, https://www.drks.de/drks_web/.

**Supplementary Information:**

The online version contains supplementary material available at 10.1007/s00417-023-06171-5.

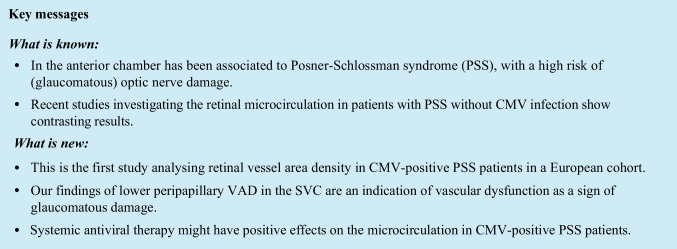

## Introduction

Cytomegalovirus (CMV) is a ubiquitous herpes virus that might affect all ocular structures with long-term persistence, leading to recurrent infections. A rare form of anterior uveitis (AU) that has been linked to a CMV infection is the Posner-Schlossman syndrome (PSS). It is also termed glaucomatocyclitic crisis and was first described by Posner and Schlossman in 1948 [1]. It mostly occurs unilaterally and is characterised by recurrent episodes of non-granulomatous AU with remarkably increased intraocular pressure (IOP) [1, 2]. Other characteristics of PSS include few keratic precipitates, the absence of iris synechia, and only mild anterior chamber reaction [3]. The aetiology of PSS is still unclear, but recent studies have shown a causal relation between PSS and CMV infection, although CMV-negative PSS has been reported [4–6]. An aqueous humour analysis is recommended to exclude other viral aetiologies, such as rubella virus (RV), herpes simplex virus (HSV), or varicella zoster virus (VZV) and to initiate adequate antiviral therapy [3, 8]. Earlier studies demonstrated a significant reduction in the recurrence rate after systemic treatment with the nucleoside analogue valganciclovir [7, 9, 10].

In the past, PSS was thought to be a more benign uveitis entity, but newer findings have shown a loss of corneal endothelial cells (CEC) and a significant thinning of the retinal nerve fibre layer (RNFL) in affected eyes [11, 12]. Furthermore, frequent recurrences of severely elevated IOP may be a risk factor for developing secondary optic neuropathy and chronic secondary glaucoma [2, 13, 14]. Interestingly, studies also suggest that CMV can cause vascular dysfunction and systemic hypertensive complications [15, 16]. Although there is an increased interest in morphologic changes in CMV-positive PSS, it remains unclear how it affects the retinal vasculature.

Optical coherence tomography angiography (OCTA) is a new non-invasive imaging method that visualises retinal blood vessels using multiple OCT scans [18]. It distinguishes blood flow from static tissue using an algorithm [19, 20]. It is a common procedure and is comparable to structural OCT measurements of retinal nerve fibre and ganglion cell thickness for differentiating glaucomatous from healthy eyes [21]. The use of OCTA to detect morphological changes in the retinal microvasculature, such as peripheral vascular dysfunction and reduced peripapillary and macular vessel area density (VAD), has been demonstrated in patients with various forms of glaucoma [19, 20, 22]. To date, three studies have investigated microvascular changes assessed by OCTA in PSS patients without evidence of CMV [23, 24].

We aimed to investigate the retinal microvasculature in CMV-positive PSS patients. Therefore, we analysed alterations of the VAD in the different retinal vascular plexuses on OCTA in PSS affected eyes in the subacute or remission phase compared to the non-affected contralateral eyes.

## Methods

### Study setting

In this monocentric, prospective case series, 31 patients with CMV-positive PSS were enrolled at the Department of Ophthalmology of the Charité–Universitätsmedizin Berlin between February 2020 and January 2022. All participating patients gave written informed consent. The study complies with the ethical principles for medical research as outlined in the Declaration of Helsinki and approval was given by the local ethics committee of Charité–Universitätsmedizin Berlin (EA4/168/17).

### Study population

The inclusion criteria were patients aged 18 years or older with a confirmed diagnosis of CMV-positive PSS. According to the updated classification criteria for CMV-positive AU, we used the following diagnostic criteria (DC): (1) proof of CMV-infection in an aqueous humour sample, (2) at least one transient episode of unilateral elevated intraocular pressure (IOP) over 21 mmHg, (3) low-grade anterior uveitis (no retinitis), (4) no iris synechia, (5) no or minimal cells in the anterior chamber, and (6) an exclusion of sarcoidosis and syphilis [3]. We excluded patients with (1) detection of RV, HSV, or VZV in the aqueous humour, (2) refractive errors of the sphere or the cylinder ≥ − 3.50 dioptres, (3) a history of other retinal diseases (diabetic retinopathy, age-related macular degeneration), and (4) amaurosis in the contralateral eye.

### Intraocular cytomegalovirus detection (in aqueous humour and blood serum)

An anterior chamber tap of approximately 100 μl was performed on patients on eligible patients, followed by a blood sampling. The samples were cryoconservated at – 20 °C and then defrosted and centrifuged at 150*g* for 1 min at room temperature for further analysis. They were analysed at the laboratory of the Charité Eye Clinic using a modified micro-ELISA technique (Enzygnost, Dade Behring Marburg, Germany) to detect CMV, HSV, VZV, or RV-specific IgG antibodies. The Goldmann-Witmer coefficient (GWC) was utilised to calculate the antibody-index (AI). A sample was tested as positive if the AI was ≥ 3.0 [5, 11, 26].

The study used the established GWC method to analyse aqueous humour, which is sensitive and specific for CMV infection within a wider diagnostic window and is less dependent on the time of sampling [6, 27, 28]. Patients with positive anterior chamber tap results for HSV, VZV, and RV were excluded to ensure homogeneity and avoid overlap with other diseases.

### Ophthalmological examination

All patients underwent a comprehensive ophthalmological examination including best-corrected visual acuity in decimal, IOP measurement using Goldmann applanation tonometry, slit-lamp examination, dilated fundus examination, and assessment of the CEC performed by a specular microscope (NidekCEM-530, NIDEK Co., Ltd. Japan). All data was obtained following approval of the diagnosis of CMV-positive PSS. We performed a baseline bilateral standard automated 66-points perimetry visual field (VF) test using the Oculus Twinfield (OCULUS, Wetzlar, Germany), and measured blood pressure and pulse rate on the same day before the imaging. Ophthalmologic and medical histories were recorded, such as: arterial hypertension, diabetes, cardiovascular or inflammatory diseases, the number of anti-glaucomatous medications, and the current SAT.

### Optical coherence tomography (OCT) angiography (OCTA)

We used the Heidelberg Engineering Spectralis for OCT and OCTA imaging (Heidelberg Engineering GmbH, Heidelberg, Germany, software version1.10.4.0). Image quality ranged from 20 to 50 [30], and the integrated OCTA software also reduced artefacts [31].

**Fig. 1:**
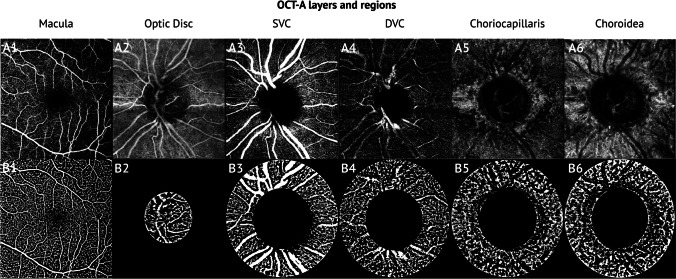
Example pictures of the OCTA en-face images: A1—macula, A2—optic disc, A3—superficial vascular complex, A4—deep vascular complex, A5—choriocapillary layer, A6—choroidal layer. The corresponding binarized images are represented in B1 to B6 with a peripapillary circle of 750 μm

The following parameters were chosen for further analysis:

1. **OCT**
  *Retinal nerve fibre layers (RNFL)*
  The RNFL is defined as the layer between the internal limiting membrane (ILM) and the ganglion cell layer that was automatically measured by the device software. The peripapillary region was divided into four quadrants: superior, inferior, temporal, and nasal. The device software calculates the average value of all quadrants (global RNFL thickness).
  *Foveal avascular zone (FAZ)*
  The foveal FAZ in mm^2^ was automatically calculated by the Heidelberg Engineering Spectralis device software after it had been delineated by the investigator (PH).
  *Retinal thickness*
  The OCT recordings used a 20° × 20° volume scan, 49 sections at a distance of 122 μm. The device software automatically calculated the average retinal thickness in μm as the distance from the retinal pigment epithelium (RPE) to the ILM at the highest point within a circle of 1 mm radius centred on the fovea. We also recorded the thickness of the superior, inferior, temporal, and nasal subfield.
2. **OCTA**
  According to a review by the American Academy of Ophthalmology, OCTA has a diagnostic ability (area under the receiver operating characteristic curve-AUC) that is comparable to structural OCT measurements of retinal nerve fibre and ganglion cell thickness [21]. Based on the consistently good AUC values for (peri-)papillary and macular VAD in the differentiation of glaucomatous from healthy eyes [19, 32–34], we assume that the analysis of VAD by OCTA is a valid method for our study.
  *Vessel area density (VAD)*
  The ImageJ software (version 1.53a) was used for all computations of the VAD [36]. First, the optic nerve head (ONH) margin, defined by the end of the Bruch’s membrane, was manually delineated by analysing the correlating OCT B-scans. A template of the ONH was placed next to the en-face image. Then, the OCTA scans were opened in ImageJ and transformed into 8-bit images. By drawing a circle around the ONH using the oval selection tool and the template next to the en-face image, the total pixel count (TPC) of the optic disc area could be measured.
  Afterwards, the Niblack’s method was utilised to binarize the image with a defined threshold of 255 [37]. The Niblack local threshold binarization algorithm improves consistency in OCTA image analysis, achieving high intraclass correlation coefficients [38, 39] and consistent results for FAZ and analysis without pre-processing [40].
  The resulting black and white pixels were measured and named as ‘white pixel count’ (WPC) and divided by the TPC. This quotient is equivalent to the VAD [41].
  This method was used to measure the VAD of the optic disc region, the peripapillary superficial vascular complex (SVC), the deep vascular complex (DVC), the choriocapillaris layer (CCL), the peripapillary choroidal layer (CL), and the SVC of the macular region. The retinal layer segmentation was automatically calculated by the OCTA viewing module 6.6.0.1 and was applied to the en-face slabs for each vascular plexus. A circular area with a radius of 750 μm extending outward from the ONH was determined as a peripapillary region (Fig. 1) [37].

### Treatment

During the acute episode, patients received topical IOP-lowering medication and topical steroids. Additionally, oral acetazolamide was initiated in some cases. After confirmation of CMV, patients were started on antiviral therapy (valganciclovir, Valcyte®). This consisted of 900 mg twice daily for two weeks, followed by 450 mg twice daily for at least three months. No patient was receiving topical or systemic steroids at the time of the examination.

### Main outcome measures

Our primary outcome measures were the differences between the VAD in the macular, the optic disc, and the peripapillary region in PSS affected eyes versus the non-affected fellow eyes.

Secondary outcome measures were the differences in RNFL, MD, macular thickness, IOP, cup-disc-ratio (CDR), CEC density, the number of anti-glaucomatous drugs, and FAZ.

### Statistical methods

Statistical analysis was performed using IBM SPSS Statistics (IBM Corp. 2019. IBM SPSS Statistics for Windows, version 26.0. Armonk, NY: IBM Corp). A sample size calculation assumed a mean VAD in the SVC, based on previously published data from our study group using the same approach and matched-pair analysis [37]. Based on a power of 90% and an alpha level of 5%, we estimated that a sample size of 16 patients would allow the detection of a 1.0% difference in VAD. For continuous variables, descriptive statistics were expressed as mean ± standard deviation (SD). Due to the small sample size, the non-parametric Wilcoxon test was used. Correlation analysis between RNFL thickness or MD and VAD was performed using Spearman’s correlation coefficient. Possible clinical risk factors (< or ≥ 1year disease duration, with or without SAT) were tested for bivariate association with the outcome parameters of the diseased eye. Initial PSS onset was defined as the first recorded IOP elevation. For the primary outcomes, differences were considered statistically significant if *p* values were less than 0.05. *p* values were adjusted for multiple testing using the Benjamini and Hochberg procedure. *p* values for secondary endpoints were not adjusted for multiple testing because they do not allow for confirmatory conclusions.

## Results

In total, 50 eyes of 25 patients with a mean age of 47 ± 13 years were included. Six patients were excluded. Four patients did not meet the IC or exclusion criteria (EC) for different reasons: one because the aqueous humour sample was negative for CMV and positive for VZV (DC 1), one because of iris synechiae and no evidence of IOP elevation (DC 2 and 4), one because of refractive errors greater than 3.50dpt in the sphere (EC 2), and one because of amaurosis in the contralateral eye (EC 4). Two patients had to be excluded because OCTA-imaging could not be performed or was of insufficient quality (image quality < 20). Baseline characteristics are presented in Table 1. Nineteen patients (76%) were receiving SAT at the time of the examination or had received SAT in the past. Six patients (24%) did not receive SAT due to side effects or family planning. Disease duration in our cohort ranged from 2 weeks to 22 years.

**Table 1:** Demographic data and clinical characteristics

Clinical parameters	
Number of patients	25
Number of eyes	50
Female sex—no. (%)	5 (20%)
Mean age in years ± SD	47 ± 13
Diabetes mellitus—no. (%)	3 (12%)
Arterial hypertension—no. (%)	7 (28%)
RR systolic in mmHg ± SD	129 ± 16
RR diastolic in mmHg ± SD	86 ± 11
MAP in mmHg ± SD	101 ±1 2
Pulse in no./min ± SD	69 ± 12
Patients treated with oral valganciclovir—no. (%)	19 (76%)

*RR*, Riva-Rocci blood pressure; *MAP*, mean arterial pressure

The mean arterial pressure is the diastolic pressure added to one-third of the difference between systolic and diastolic pressure in mmHg

The ophthalmological findings are presented in Table 2. We found differences in the highest IOP, the VF MD, the RNFL thickness in three quadrants, the CDR, the number of anti-glaucomatous drugs, and the corneal endothelial cell density between the PSS affected and the contralateral eye. There was no difference in the current IOP and macular retinal thickness between two eyes, except in the nasal quadrant.

**Table 2:** Secondary outcomes: diagnostic measurements in eyes affected by Posner-Schlossman syndrome versus the nonaffected fellow eyes

Parameters	Affected eye	Fellow eye	*p* value	Mean difference with 95%CI
Visual acuity in LogMAR	0.15 ± 0.40	0.04 ± 0.18	0.316^a^	0.11 [− 0.05; 0.27]
Highest IOP in mmHg	44.1 ± 11.5	16.8 ± 2.8	**< 0.001**^**a**^	**27.2 [23.0; 31.8]**
IOP in mmHg	16.7 ± 6.0	14.6 ± 1.8	0.262^a^	1.9 [− 0.3; 4.4]
VF MD in dB	− 4.8 ± 7.9	− 1.7 ± 3.2	**0.029**^**a**^	**− 3.1 [− 6.1; − 0.1]**
CDR	0.48 ± 0.29	0.39± 0.22	**0.014**^**a**^	**0.09 [0.02; 0.16]**
Anti-glaucoma eye drops – no.	1.6 ± 1.5	0.6 ± 1.4	**0.002**^**a**^	**1.0 [0.5; 1.5]**
CEC-density–cells/mm^2^	2416 ± 486	2690 ± 352	**0.001**^**a**^	**− 276 [− 542; − 10,3]**
RNFL thickness in μm				
Average	80.1 ± 22.6	92.8 ± 14.4	**0.002**^**a**^	**− 12.7 [− 20.4; − 4.9]**
Superior	94.0 ± 28.1	110.3 ± 20.5	**0.004**^**a**^	**− 16.3 [− 25.9; − 6.6]**
Inferior	105.7 ± 35.0	124.8 ± 22.3	**0.019**^**a**^	**19.1 [− 12.8; − 1.4]**
Temporal	62.8 ± 19.8	70.4 ± 14.3	0.069^a^	− 7.6 [− 14.1; − 0.9]
Nasal	58.1 ± 16.6	65.8 ± 14.5	**0.015**^**a**^	**− 7.7 [− 13.5; − 1.8]**
SSI	29.2 ± 4.0	29.6 ± 3.5	0.297^a^	− 0.4 [− 1.3; 0.5]
Retinal thickness in μm				
Average	333.3 ± 26.6	338.2 ± 16.8	0.241^a^	− 4.9 [− 12.0; 2.3]
Superior	336.0 ± 28.2	341.0 ± 17.7	0.171^a^	− 5.0 [− 13.1; 3.1]
Inferior	333.2 ± 28.1	338.9 ± 19.7	0.258^a^	− 5,7 [− 12.8; 1.4]
Temporal	330.2 ± 27.6	329.4 ± 16.5	0.845^a^	0.8 [− 8.5; 10.3]
Nasal	334.9 ± 25.9	343.4 ± 16.1	**0.022**^**a**^	**− 8.5 [− 14.8; − 2.1]**
SSI	29.9 ± 4.0	31.4 ± 5.1	**0.038**^**a**^	− 1.5 [− 3.2; 0.2]
OCTA parameters				
FAZ in mm^2^	0.45 ± 0.20	0.44 ± 0.16	0.954^a^	0.01 [− 5.8; 6.7]
Macula OCTA SSI	34.1 ± 4.1	34.2 ± 3.6	0.708^a^	− 0.1 [− 1.3; 1.0]
Peripapillary OCTA SSI	32.7 ± 3.7	34.0 ± 2.9	0.079^a^	− 1.3 [− 2.7; 0.8]

*IOP*, intraocular pressure; *VFMD*, visual field mean deviation; *CDR*, cup disc ratio; *CEC*, corneal endothelial cell; *RNFL*, retinal nerve fibre layer; *SSI*, signal strength index; *CI*, confidence interval; *OCTA*, optical coherence tomography angiography; *FAZ*, foveal avascular zone

Parameters are shown as mean ± standard deviation

^a^Wilcoxon-test

Values in bold indicate *p*-values < 0.05

Table 3 summarises the results of the perfusion parameters assessed by the OCTA. There were no significant differences in the macular and optic disc vascular parameters between both groups. Only the superficial vascular complex on the peripapillary regions showed a significant decrease in the VAD in the PSS eye (46.1 ± 9.3% versus 50.1 ± 6.3%, *p* = 0.008, adjusted *p* = 0.048). All the deeper layers (DVC, CCL, CL) did not differ between the two groups.

**Table 3:** Primary outcomes: optical coherence tomography angiography macular, papillary, and peripapillary vessel area density parameters in eyes affected by Posner-Schlossman syndrome versus the nonaffected fellow eyes

Parameters	Affected eye	Fellow eye	*p* value; adjusted *p* value	Mean difference with 95%CI
Macula OCTA				
VAD in %	34.1 ± 3.9	35.1 ± 2.5	0.143^a^; 0.286^b^	1.0 [− 2.0; 0.1]
Optic disc OCTA				
VAD in %	38.6 ± 2.7	39.2 ± 2.3	0.276^a^; 0.414^b^	0.6 [− 1.6; 0.4]
Peripapillary OCTA				
VAD SVC in %	46.1 ± 9.3	50.1 ± 6.3	**0.008**^**a**^**; 0.048**^b^	**4.0 [− 6.5; − 1.4]**
VAD DVC in %	33.4 ± 2.7	34.0 ± 2.1	0.288^a^; 0.346^b^	0.6 [− 1.5; 0.3]
VAD CCL in %	34.3 ± 2.2	34.0 ± 1.9	0.353^a^; 0.353^b^	0.7 [− 0.4; 1.0]
VAD CL in %	35.8 ± 1.8	34.9 ± 1.9	0.065^a^; 0.195^b^	1.1 [− 0.0; 1.8]

*OCTA*, optical coherence tomography angiography; *VAD*, vessel area density; *SVC*, superficial vascular complex; *DVC*, deep vascular complex; *CCL*, choriocapillaris layer; *CL*, choroidal layer; *CI*, confidence interval

Values are shown as mean ± standard deviation

^a^Wilcoxon-test

^b^adjusted p values by Benjamini and Hochberg procedure

Values in bold indicate *p*-values < 0.05

In Table 4, we analysed the correlation between the clinical findings (MD of the VF, RNFL) and the vessel perfusion parameters assessed by OCTA. We found a significant correlation between the MD and the following parameters: the VAD of the macular, the optic disc, the peripapillary SVC, the peripapillary DVC, and the FAZ. Correlation analyses of the RNFL showed similar results with a significant correlation of the RNFL with the VAD of the macula, the optic disc, the peripapillary SVC, and the FAZ. The deeper peripapillary layers (CCL, CL) showed no correlation.

**Table 4:** Correlation analysis of the mean deviation in visual field testing or the retinal nerve fibre layer and the optical coherence tomography angiography parameters in eyes affected by Posner-Schlossman syndrome

Clinical parameters	Macula VAD	FAZ	Optic disc VAD	SVC VAD	DVC VAD	CCL VAD	CL VAD
MD	0.451 ***p*** **= 0.001**	− 0.293 ***p*** **= 0.043**	0.512 ***p*** **< 0.001**	0.430 ***p*** **= 0.002**	0.462 ***p*** **< 0.001**	0.161 *p* = 0.275	− 0.023 *p* = 0.879
RNFL	0.553 ***p*** **< 0.001**	− 0.283 ***p*** **= 0.047**	0.292 *p* **= 0.039**	0.723 ***p*** **< 0.001**	0.151 *p* = 0.295	− 0.215 *p* = 0.134	0.433 ***p*** **= 0.002**

*MD*, mean deviation; *RNFL*, retinal nerve fibre layer thickness; *VAD*, vessel area density; *FAZ*, foveal avascular zone; *SVC*, superficial vessel complex; *DVC*, deep vessel complex; *CCL*, choriocapillaris layer, *CL*, choroidal layer

All values are shown as rho value and *p* value

Spearman correlation coefficient

Values in bold indicate *p*-values < 0.05

Through analysing the association between the results of the affected eye and the disease duration, we detected a dependence of the mean RNFL on the disease duration (*p* = 0.037; duration < 1 year: 87.1 ± 20.0 μm, duration ≥ 1 year: 78.0 ± 24.0 μm). The subanalysis of patients with disease duration < 1 year is shown in Table 5. There were no significant differences in VAD of all layers between the affected and non-affected eye. Table 6 shows the subanalysis of patients with disease duration ≥ 1year. The superficial vascular complex in the peripapillary regions showed a significant decrease in VAD in the PSS eye (44.1 ± 10.3% versus 49.6 ± 7.4%, *p* = 0.006, adjusted *p* = 0.036). All other layers (DVC, CCL, CL, macula, and ONH) did not differ between affected and non-affected eyes.

**Table 5:** Primary outcomes: optical coherence tomography angiography macular, papillary, and peripapillary vessel area density parameters in eyes affected by Posner-Schlossman syndrome versus the nonaffected fellow in patient with a disease duration less than 1 year

Parameters disease duration less than 1 year (*n* = 8)	Affected eye	Fellow eye	*p* value; adjusted *p* value
Macula OCTA			
VAD in %	35.7 ± 2.5	35.9 ± 1.1	0.575^a^; 0.690^b^
Optic disc OCTA			
VAD in %	39.2 ± 3.0	38.4 ± 2.0	0.263^a^; 0.395^b^
Peripapillary OCTA			
VAD SVC in %	50.5 ± 4.5	51.0 ± 3.3	1.0^a^; 1.0^b^
VAD DVC in %	34.4 ± 1.0	33.4 ± 1.5	0.093^a^; 0.558^b^
VAD CCL in %	34.8 ± 2.3	33.6 ± 1.8	0.123^a^; 0.369^b^
VAD CL in %	35.9 ± 2.1	34.1 ± 1.9	0.161^a^; 0.322^b^

*OCTA*, optical coherence tomography angiography; *VAD*, vessel area density; SVC, superficial vascular complex; *DVC*, deep vascular complex; *CCL*, choriocapillaris layer; *CL*, choroidal layer

Values are shown as mean ± standard deviation

^a^Wilcoxon-test

^b^adjusted p values by Benjamini and Hochberg procedure

**Table 6:** Primary outcomes: optical coherence tomography angiography macular, papillary, and peripapillary vessel area density parameters in eyes affected by Posner-Schlossman syndrome versus the nonaffected fellow in patient with a disease duration of 1 year or more

Parameters disease duration 1 year or more (*n* = 17)	Affected eye	Fellow eye	*p* value; adjusted *p* value
Macula OCTA			
VAD in %	33.4 ± 4.3	34.6 ± 2.9	0.068^a^; 0.102^b^
Optic disc OCTA			
VAD in %	38.2 ± 2.7	39.5 ± 2.4	**0.039**^**a**^; 0.078^b^
Peripapillary OCTA			
VAD SVC in %	44.1 ± 10.3	49.6 ± 7.4	**0.006**^**a**^**; 0.036**^**b**^
VAD DVC in %	32.9 ± 3.1	34.3 ± 2.2	**0.025**^**a**^; 0.075^b^
VAD CCL in %	34.0 ± 2.2	34.2 ± 2.0	0.943^a^; 0.943^b^
VAD CL in %	35.8 ± 1.7	35.4 ± 1.7	0.163^a^; 0.196^b^

*OCTA*, optical coherence tomography angiography; *VAD*, vessel area density; *SVC*, superficial vascular complex; *DVC*, deep vascular complex; *CCL*, choriocapillaris layer; *CL*, choroidal layer

Values are shown as mean ± standard deviation

^a^Wilcoxon-test

^b^adjusted *p* values by Benjamini and Hochberg procedure

Values in bold indicate *p*-values < 0.05

Additionally, patients receiving SAT showed a significantly better VAD of peripapillary CCL (*p* = 0.001, no therapy: 31.4 ± 1.9%, SAT: 35.0 ± 1.6%) and peripapillary CL (*p* = 0.009, no therapy: 34.2 ± 0.3%, SAT: 36.3 ± 1.8%), while all other parameters did not differ (supplementary data).

## Discussion

In this prospective observational case series, the VAD in the peripapillary SVC is significantly reduced in CMV-positive PSS-affected eyes, when compared to the non-affected eyes in the subacute or remission phase. This is consistent with a decrease in RNFL thickness and VF MD in PSS-affected eyes. Additionally, the reduction of RNFL seems to be dependent on the duration of the disease. The deeper layers showed no significant differences between CMV-positive PSS-affected and non-affected eyes. However, there might be an impact of the antiviral therapy on the VAD of the peripapillary CCL and CL layer.

The VAD in the peripapillary SVC is significantly reduced in CMV-positive PSS-affected eyes compared to non-affected eyes. Chen et al. reported comparable results in PSS-patients [23], while the study by Liu et al. in newly diagnosed PSS-patients without proof of CMV, where OCTA images were taken during the first acute attack and 1 week after treatment, presented opposite results [24]. However, this study is not comparable to ours as we investigated patients in the subacute or remission phase. Similar observations to our results have been reported for secondary IOP elevation in patients with unilateral FUS [42]. Our results are consistent with earlier research in patients with different forms of glaucoma, such as primary open angle glaucoma (POAG), acute primary angle closure (APAC), pseudoexfoliation glaucoma (PXG), and normal-tension glaucoma (NTG) [43–47]. Liu et al. demonstrated that the peripapillary SVC is particularly affected in patients with perimetric glaucoma, and the authors hypothesised that this change is a sign of glaucomatous optic disc damage [49]. Additionally, Wang et. al. found reduced peripapillary VAD in patients with APAC even without measurable structural differences (RNFL thickness and ganglion cell complex thickness) compared to the non-affected eye [50]. Another study in patients with APAC indicated that a longer duration of an acute attack was correlated with reduced peripapillary VAD [51], and Moghimi et al. described a progressive decrease in circumpapillary VAD over the first 6 weeks after the initial insult [52].

The current research demonstrated no significant differences between the VAD of the optic disc and the deeper peripapillary layers. This is consistent with another study that found no differences in the VAD of the DVC in glaucomatous eyes compared to healthy controls [49].

No significant differences were found in macular thickness and the VAD of the macular SVC between the two groups. This is in line with the study of Liu et al., which also reported no changes in the VAD of the macular SVC [24]. However, in PSS eyes without confirmed CMV, two studies found a reduced superficial macular VAD compared to the fellow eye and a healthy control group [23, 25].

In contrast to our study, which captured OCTA images during the remission phase, in the study of Chen et al., OCTA images were taken closer to the acute phase (within one to seven days afterwards) [23]. This may explain the lower macular superficial VAD in PSS eyes, which probably recovers during remission. Trabeculectomy showed similar reversal effects on the VAD in POAG patients, with postoperative increase in VAD correlating to higher preoperative IOP and greater IOP reduction [53]. In contrast, Liu et al. found no changes in the VAD of the macular and peripapillary SVC despite high IOP reduction [24]. In this study, selected PSS patients displayed no changes in VF and RNFL thickness. These patients without glaucomatous damage might have strong retinal autoregulation to maintain the blood flow in the peripapillary and macula region under elevated IOP, while patients with mild and advanced glaucomatous damage might not.

Another possible explanation of the missing differences in the macular parameters in our study could be the beneficial effect of therapy with valganciclovir on the overall retinal and choroidal microvasculature. We found a better VAD of the CCL and CL in patients receiving antiviral therapy compared to those refusing antiviral treatment. Contrary to our study, in which we treated more than three-quarters of the patients with valganciclovir, none of the patients in the studies on PSS and microvascular changes on OCTA were treated with antiviral medication [23, 24]. Additionally, lowering IOP and improving retinal blood flow by an anti-glaucomatous therapy of the affected eye may also be a reason for a lack of differences in this layer [54, 55].

Additionally, our results might be explained by the chronological progression of glaucoma, as seen in other glaucoma types (POAG, PXG, APAC), where disease severity correlates with macular VAD reduction [45, 56, 57]. The SVC seems to be more affected than the DVC, like the peripapillary region [59]. Initial and mild glaucomatous damage is indicated by peripapillary VAD and RNFL changes, as supported by our data [60, 61]. Advanced stages may result in VF defects and macular VAD reduction. In our cohort, the glaucomatous damage was relatively mild, with minor VF alterations and small CDR differences. We cannot rule out the possibility that macular damage might be detectable in more advanced stages. Our thesis is supported by significant correlations between peripapillary, papillary, and macular VAD and reductions in RNFL thickness and VF MD [62]. Furthermore, the RNFL appears to be dependent on the duration of disease. Research on glaucoma patients with POAG showed similar results, particularly towards the macular VAD, correlating significantly with a reduction in RNFL thickness and an increase in VF MD [43, 58, 63, 64]. The results of Liu et al. confirm this assumption. In affected, newly diagnosed eyes, no glaucomatous damage, and no changes in VAD were present. Our study included patients with different disease duration. Some of them had no glaucomatous changes. Other patients, some with partially longer disease courses, were more likely to have mild or advanced glaucomatous damage, such as reduced RNFL thickness and consecutive first changes in VAD, as shown in our subgroup analysis.

In line with other studies on PSS, we found a higher proportion of males, an increased maximum IOP, an average age of 47 ± 13 years, differences in MD, CDR, and the amount of anti-glaucomatous agents [66]. As reported previously, a loss of CEC and RNFL thickness was also detectable in our patients [11].

It is unclear whether the glaucomatous damage in PSS is due to elevated IOP or due to CMV infection and vascular dysfunction. Experimental trials have shown that CMV is associated with immune activation in T-cells. This can cause atherosclerotic progression and chronic inflammation, leading to endothelial damage and vascular dysfunction [16, 17]. Infection of vascular endothelial cells and macrophages appears to be especially important for CMV latency and possible reactivation in the host. Furthermore, their finding of persistent ocular inflammation following CMV infection in a mouse model of an immunocompetent host underscores the need to consider CMV as a pathogen capable of inducing long-term inflammatory sequelae in the eye, including the neural retina [67]. In another experimental murine model, CMV infection in the eye is first detected in endothelial cells of the iris and causes inflammation and a latent infection of the retinal pigment epithelium [68]. These results may support our findings and hypothesis of vascular impairment in ocular tissue due to CMV infection. Our study allows only speculation about vascular dysfunction, as we did not compare our results to healthy controls, and the previously mentioned studies were not conducted in humans. However, we could detect differences in microcirculation between affected and non-affected eyes that might play a role in the development of glaucomatous damage.

In the Asian population, approximately 50% of PSS patients were identified to be CMV-positive [6]. To date, there are no comparable studies in a European cohort, but we suspect that the incidence of CMV-infection is lower in Germany. To analyse the data in a more homogeneous group, we based our study on CMV-positive PSS only. There are several reasons for this, as the clinical characteristics of CMV-positive and CMV-negative PSS appear to be similar. Nonetheless, CMV-positive PSS was associated with greater CEC loss and a larger CDR [69]. Furthermore, the number of eyes treated surgically was higher in the virus-proven group [70].

Currently, there is no standardised guideline for the optimal treatment of CMV-associated PSS, but patients might benefit from antiviral therapy [9, 10]. Previous studies reported a good response to valganciclovir, a lower recurrence rate, a reduction in glaucoma severity, and reduced need for glaucoma surgery [7, 10, 71]. Unfortunately, additional glaucoma surgery was required in some cases, especially when the disease had been present for more than 5 years [7, 70, 71]. Our results support these findings, as trabeculectomy was performed in two of our patients with more than 5 years of disease.

We are aware of certain limitations of this study. Firstly, the sample size was relatively small due to the rare occurrence and the often-asymptomatic clinical characteristics. Secondly, OCTA imaging was performed during the subacute or remission phase. This was related to the often-limited image quality due to acute IOP elevation and corneal oedema, which did not meet our quality requirements. Additionally, the RNFL examination may be misleading in the first days or weeks after the attack due to oedema [72]. Therefore, we recommend OCT and OCTA examinations during the remission phase. In addition, some of our patients received topical anti-glaucomatous therapy, which may affect retinal blood flow. In particular carbonic anhydrase inhibitors such as dorzolamide or brinzolamide were found to improve retinal blood flow [54, 55]. Accordingly, anti-glaucomatous medication should not lead to a reduction of superficial peripapillary VAD in the affected eye but are possible the reason for the lack of differences in the other layers and regions as discussed before.

Furthermore, we only included patients with confirmed CMV-positive PSS, and the antibody analysis took about 1 to 2 weeks. Additionally, the results covered different disease durations, which limits generalisation. However, longer disease duration correlated with a reduced VAD in the peripapillary SVC. Data collection relied on patients’ recall of their first attack, which may not be accurate as it remains unclear whether patients had previous undetected attacks. Thirdly, we only examined the retinal vasculature at a single time point. Longitudinal follow-up with OCTA would provide more reliable information on disease progression. The investigation of individuals with different disease progression partially addressed this limitation. However, larger longitudinal studies with a control group are needed to investigate long-term changes in the retinal microvasculature. Fourth, our study focused only on CMV-positive PSS. This limits the generalizability to all PSS patients.

## Conclusion

This study is the first to investigate the peripapillary microvasculature in CMV-positive PSS in Europe. Challenges for future management include high IOP, glaucomatous damage, recurrence rate, and optimal treatment. More than three-quarters of our patients received SAT, which may benefit the retinal microvasculature. A more standardised VAD analysis with defined assessment of retinal layers is needed in the future. Nonetheless, lower peripapillary VAD in the SVC may indicate vascular dysfunction and impaired blood flow to the ONH. Therefore, we suggest OCTA as an additional tool for the follow-up of patients with PSS to detect changes in VAD that might lead to glaucomatous damage.

### Supplementary information

ESM 1 (DOCX 22 kb)
