# Supplementary material for: Cytomegalovirus-positive Posner-Schlossman syndrome: to compare differences in retinal vessel area density between the affected and non-affected eye using optical coherence tomography angiography
Source: Graefes Arch Clin Exp Ophthalmol. 2023 Jul 22;261(11):3263–74. doi: 10.1007/s00417-023-06171-5 (PMC10587322; doi:10.1007/s00417-023-06171-5)
Supplement: Supplementary file 1 — (DOCX 22 kb) [file 417_2023_6171_MOESM1_ESM.docx]

**Supplementary Information**

**Cytomegalovirus-positive Posner-Schlossman Syndrome:**

**To compare differences in retinal vessel area density between the affected and non-affected eye using Optical Coherence Tomography Angiography**

Graefe's Archive for Clinical and Experimental Ophthalmology

Patricia Hülse^1^, Emanuel Reitemeyer^1^, Anne Rübsam^1^, Uwe Pleyer^1^, Anna-Karina B. Maier ^1^

^1^ Charité – Universitätsmedizin Berlin, corporate member of Freie Universität Berlin, Humboldt Universität zu Berlin, and Berlin Institute of Health, Berlin, Germany, Department of Ophthalmology, Augustenburger Platz, 13353 Berlin, Germany

***Corresponding author: Anna-Karina B. Maier M.D**

E-mail: [anna-karina.maier@charite.de](mailto:anna-karina.maier@charite.de)

**Table S1** Median, interquartil range and range of secondary outcomes: diagnostic measurements in eyes affected by Posner-Schlossman Syndrome versus the nonaffected fellow eyes

| **Parameters** | **Affected Eye**  **median** | **Interquartil**  **range** | **Range** | **Fellow eye**  **median** | **Interquartile**  **range** | **Range** |
| --- | --- | --- | --- | --- | --- | --- |
| Visual acuity in LogMAR (±SD) | 0.00 | [0.00;0.10] | [-0.10;1.50] | 0.00 | [0.00;0.00] | [-0.10;0.80] |
| Highest IOP in mmHg (±SD) | 41 | [34;54] | [23;64] | 16 | [15;19] | [11;24] |
| IOP in mmHg | 15 | [14;19] | [9;40] | 14 | [14;16] | [11;18] |
| VF MD in dB | -1.85 | [-4.76;-2.23] | [-26.70;0.60] | -0.45 | [-2.13;-0.03] | [-12.90;0.70] |
| CDR | 0.40 | [0.20;0.675] | [0.10;1.00] | 0.30 | [0.20;0.50] | [0.10;0.90] |
| Anti-glaucoma eye drops – no. | 1 | [0;3] | [0;4] | 0 | [0;0] | [0;4] |
| CEC-density – cells/mm^2^ | 2494 | [2239;2777] | [1142;3247] | 2661 | [2515;2952] | [1805;3362] |
| **RNFL thickness in μm** |  |  |  |  |  |  |
| Average | 87 | [61;100] | [32;108] | 95 | [88;102] | [38;109] |
| Superior | 103 | [73;115] | [38;144] | 112 | [102;125] | [45;137] |
| Inferior | 127 | [68;136] | [40;144] | 127 | [116;136] | [49;172] |
| Temporal | 63 | [49;76] | [27;97] | 71 | [60;78] | [37;101] |
| Nasal | 60 | [48;70] | [19;86] | 68 | [55;76] | [23;87] |
| SSI | 30 | [26;32] | [22;35] | 29 | [28;32] | [22;36] |
| **Retinal thickness in μm** |  |  |  |  |  |  |
| Average | 337 | [311;337] | [284;377] | 337 | [329;354] | [289;365] |
| Superior | 338 | [316;354] | [277;393] | 342. | [336;353] | [281;366] |
| Inferior | 341 | [305;357] | [278;378] | 342 | [328;354] | [282;374] |
| Temporal | 334 | [310;347] | [285;403] | 332 | [316;344] | [293;355] |
| Nasal | 340 | [315;356] | [287;371] | 340 | [337;360] | [299;366] |
| SSI | 31 | [27;33] | [21;35] | 32 | [28;34] | [21;41] |
| **OCTA parameters** (±SD) |  |  |  |  |  |  |
| FAZ in mm^2^ | 0.410 | [0.295;0.545] | [0.120;0.910] | 0.440 | [0.325;0.450] | [0.230;0.960] |
| Macula OCTA SSI | 35 | [32;37] | [26;41] | 34 | [32;37] | [27;40] |
| Peripapillary OCTA SSI | 32 | [29;36] | [26;40] | 34 | [33;36] | [28;42] |

IOP: intraocular pressure, VF MD: visual field mean deviation, CDR: cup disc ratio, CEC: corneal endothelial cell, RNFL: retinal nerve fiber layer thickness, SSI: signal strength index, OCTA: optical coherence tomography angiography, FAZ: foveal avascular zone

**Table S2** Median, interquartil range and range of primary outcomes: optical coherence tomography angiography parameters in eyes affected by Posner-Schlossman Syndrome versus the nonaffected fellow eyes

| **Parameters** | **Affected Eye**  **Median** | **Interquartil**  **Range** | **Range** | **Fellow Eye**  **Median** | | **Interquartil**  **range** | **Range** |
| --- | --- | --- | --- | --- | --- | --- | --- |
| **Macula OCTA** |  |  |  | |  |  |  |
| VAD in % | 0.362 | [0.331;0.366] | [0.212;0.388] | | 0.357 | [0.345;0.366] | [0.262;0.377] |
| **Optic disc OCTA** |  |  |  | |  |  |  |
| VAD in % | 0.391 | [0.364;0.409] | [0.323;0.421] | | 0.394 | [0.374;0.410] | [0.338;0.427] |
| **Peripapillary OCTA** |  |  |  | |  |  |  |
| VAD SVC in % | 0.484 | [0.433;0.521] | [0.255;0.582] | | 0.509 | [0.467;0.543] | [0.302;0.585] |
| VAD DVC in % | 0.338 | [0.318;0.353] | [0.263;0.371] | | 0.343 | [0.327;0.356] | [0.298;0.373] |
| VAD CCL in % | 0.344 | [0.330;0.360] | [0.286;0.375] | | 0.343 | [0.327;0.352] | [0.3;0.376] |
| VAD CL in % | 0.355 | [0.343;0.376] | [0.332;0.387] | | 0.350 | [0.339;0.361] | [0.302;0.376] |

OCTA: optical coherence tomography angiography, VAD: vessel area density, SVC: superficial vascular complex, DVC: deep vascular complex, CCL: choriocapillaris layer, CL: choroideal layer

**Table S3** Parameters comparing patients treated with or without systemic antiviral therapy

| **Parameters** | **Patients with systemic SAT** | **Patients without systemic SAT** |
| --- | --- | --- |
| **Number of patients** | 19 | 6 |
| **Mean Age in years ±SD** | 47.5±13.9 | 46.7±11.3 |
| **Female – no.** | 4 | 1 |
| **Male – no.** | 15 | 5 |
| **Disease duration less than one year – no.** | 7 (36.8%) | 1 (16.7%) |
| **Disease duration of one year or more – no.** | 12 (63.2%) | 5 (83.3%) |

SAT: systemic antiviral therapy, SD: standard deviation, values for mean age are shown as mean ±standard deviation
